# Hospitals and the Spirit Tax

**Published:** 1916-08-26

**Authors:** 


### HOSPITALS AND THE SPIRIT TAX.

After long waiting, the promised "relief" to hospitals in respect of the tax on spirits used for medical and surgical purposes has come. It is in the shape of an annual grant of £10,000 to be made to voluntary hospitals as a compensation for the sums which these hospitals pay for spirit over and above the intrinsic cost of the same. The amount of the grant is totally inadequate for the purpose. A correspondent calls this grant an "excellent result" of Mr. Bridgeman's initiative and tenacity, but we cannot agree with him. Mr. Bridgeman's proposal—and, in fact, the Government's proposal—was to refund to hospitals the whole of the duty on spirits used in the preparation of medicines, and while the thanks of hospital authorities are due to Mr. Bridgeman for initiating a campaign which has resulted in a *douceur* of £10,000, it is absurd to call this an excellent result. Had it not been for the still unexplained action of the British Medical Association and the Pharmaceutical Society in opposing the original offer, hospitals would probably have received three times as much per annum and the concession would already have been in operation for a year.

In our issue of March 18 last, page 544, we reprobated the extraordinary attitude of the British Medical Association in assisting in the organisation of the opposition against the wise and generous proposals of the Government which has deprived the voluntary hospitals in the aggregate of thousands of pounds a year, estimated to be the equivalent of many hospital beds. This opposition and result were the measure of the incompetence of the controllers of the policy of the British Medical Association.

Ten thousand pounds represents the duty on something like 14,000 gallons of proof spirit, which is obviously only a proportion of the amount annually used in voluntary hospitals Our own estimate was that a sum in the neighbourhood of £30,000 would have covered the loss incurred annually by hospitals through the failure of the Customs and Excise authorities to distinguish between potable and medicinal spirits, and it is very difficult to understand on what facts the Committee charged with the duty of devising a scheme for relieving hospitals can have based its estimate. We hope the mischief may be remedied later on. Is it too much to hope that the new Council of the B.M.A. may reverse the evil policy of the past, and that the voluntary hospitals may be relieved from a large proportion of the tax from which the Government proposed to free them wholly.?

